# Plasma Concentrations of the Vasoactive Peptide Fragments Mid-Regional Pro-Adrenomedullin, C-Terminal Pro-Endothelin 1 and Copeptin in Hemodialysis Patients: Associated Factors and Prediction of Mortality

**DOI:** 10.1371/journal.pone.0086148

**Published:** 2014-01-22

**Authors:** Ferruh Artunc, Albina Nowak, Christian Mueller, Tobias Breidthardt, Raphael Twerenbold, Robert Wagner, Andreas Peter, Hans-Ulrich Haering, Stefan Ebmeyer, Bjoern Friedrich

**Affiliations:** 1 Department of Internal Medicine, Division of Endocrinology, Diabetology, Vascular Disease, Nephrology and Clinical Chemistry, University of Tuebingen, Tuebingen, Germany; 2 Division of Internal Medicine, Division of Nephrology, University of Zurich, Zurich, Switzerland; 3 Department of Internal Medicine, Division of Cardiology, University of Basel, Basel, Switzerland; 4 Fisher Scientific, Schwerte, Germany; 5 Dialysis Center, Leonberg, Germany; Biological Research Centre of the Hungarian Academy of Sciences, Hungary

## Abstract

Vasopressin, endothelin and adrenomedullin are vasoactive peptides that regulate vascular tone and might play a role in hypertensive diseases. Recently, laboratory assays have been developed to measure stable fragments of vasopressin, endothelin and adrenomedullin. Little is known about their diagnostic and prognostic value in hemodialysis patients. In this study, we measured the plasma concentration of copeptin, mid-regional-pro-adrenomedullin (MR-pro-ADM) and C-terminal pro-endothelin 1 (CT-pro-ET1) in stable ambulatory hemodialysis patients (n = 239) and investigated their associations with clinical factors and mortality. In all patients enrolled, the plasma concentrations of copeptin, MR-pro-ADM and CT-pro-ET1 were largely elevated with a median concentration of 132 pmol/L (interquartile range [IQR] 78–192) for copeptin, 1.26 nmol/L (IQR 1.02–1.80) for MR-pro-ADM and 149 pmol/L (IQR 121–181) for CT-pro-ET1. The plasma concentrations of all vasoactive peptide fragments correlated with time on dialysis and plasma β2-microglobulin concentration and were negatively correlated to residual diuresis. The plasma concentration of MR-pro-ADM was a strong predictor of all-cause (univariate hazard ratio for a 10-fold increase 9.94 [3.14;32], p<0.0001) and cardiovascular mortality (hazard ratio 34.87 [5.58;217], p = 0.0001) within a 3.8-year follow-up. The associations remained stable in models adjusted for dialysis specific factors and were attenuated in a full model adjusted for all prognostic factors. Plasma copeptin concentration was weakly associated with cardiovascular mortality (only in univariate analysis) and CT-pro-ET1 was not associated with mortality at all. In conclusion, vasoactive peptide fragments are elevated in hemodialysis patients because of accumulation and, most likely, increased release. Increased concentrations of MR-pro-ADM are predictive of mortality.

## Introduction

Vasopressin, endothelin and adrenomedullin are vasoactive peptides that regulate vascular tone and perfusion. After being released by vascular endothelial cells (endothelin and adrenomedullin) or pituitary gland (vasopressin), these peptides exert their actions in vascular smooth muscle cells after binding to specific G-protein coupled receptors and generating second messengers. Whereas vasopressin and endothelin lead to vasoconstriction, adrenomedullin exhibits vasodilatory effects [1]. All three have in common a short half-life of a few minutes and ex vivo instability that has precluded valid measurements in humans. To overcome this problem, laboratory assays that detect stable fragments of vasopressin, endothelin and adrenomedullin have been developed, thereby enabling applications in different patient cohorts and diseases [2]–[4]. Copeptin, the C-terminal portion of pro-vasopressin, C-terminal endothelin 1 (CT-pro-ET1) and mid-regional-pro-adrenomedullin (MR-pro-ADM) are small peptide fragments with 39, 44 and 47 amino acids in length, respectively, that are excised from large precursor peptides during posttranslational processing. They share a low molecular weight (3.4 kDa for copeptin, 5.2 kDa for CT-pro-ET1 and 5.1 kDa for MR-pro-ADM) and because they are stoichiometrically generated they can serve as a good estimate for the plasma concentrations of vasopressin, endothelin and adrenomedullin.

Accurate risk prediction is an important clinical task. In hemodialysis patients, troponins and natriuretic peptides have consistently been shown to be closely associated with patient mortality and improve risk prediction provided by clinical variables alone [5]–[7]. In this context, the search for new biomarkers reflecting different facts of cardiovascular risk is still ongoing. Among these new biomarkers, the stable fragments MR-pro-ADM, CT-pro-ET1 and copeptin are promising candidates to further improve clinical risk assessment. Interestingly, pilot studies by our group and others [8]–[12] have shown that in some settings, these novel tools even outperform natriuretic peptides [8]; [9]; [12]. However, little is known in hemodialysis patients about the performance and significance of measuring the plasma concentration of MR-pro-ADM, CT-pro-ET1 and copeptin. Two recent studies have shown that increased MR-pro-ADM and copeptin levels were associated with reduced survival [13]; [14], however, data about the prognostic value of CT-pro-ET1 are not available.

In our study, we analyzed the factors influencing the plasma concentration of copeptin, MR-pro-ADM and CT-pro-ET1 in stable ambulatory hemodialysis patients and analyzed the prognostic value of each vasoactive peptide during a 3.8-year follow-up.

## Methods

### Patients and cohort

This prospective multicenter study included stable ambulatory hemodialysis patients from four dialysis centers in Southwest Germany between September 2009 and April 2010. Patients were included after they provided written informed consent and when there was no evidence of an acute illness, a cardiac event or a procedure within the previous two months. Patients with cardiac diseases leading to increased cardiac biomarkers concentration (troponin, natriuretic peptides) independent of ESRD, such as amyloidosis were excluded. The study was approved by the local ethics committee of the University of Tuebingen (191/2009BO2).

### Laboratory assays

Plasma concentration of copeptin, MR-pro-ADM and CT-pro-ET1 were measured in three independent samples taken within 2 weeks, each one taken to the start of a dialysis session. Blood was collected in lithium-heparinized tubes (Sarstedt, Nuembrecht, Germany), cooled at 4°C and centrifuged within 4 hours; the sera were stored at −80°C for further analysis. The plasma concentrations of copeptin, MR-pro-ADM and CT-pro-ET1 were measured using an automated immunoluminometric assay on a Kryptor system (B.R.A.H.M.S AG, Henningsdorf, Germany) as described in [2]–[4]. In healthy persons, the normal range defined as the span between the 2.5^th^ and 97.5^th^ percentile was 1.7–11.3 pmol/L for copeptin [3], 0.17–0.49 nmol/L for MR-pro-ADM [2] and 24.8–66.6 pmol/L for CT-pro-ET1 [4].

The plasma concentrations of high sensitive troponin T were measured using an automated Roche assay on an Elecsys 2010 system (Roche, Basel, Switzerland). Plasma concentrations of NT-pro-BNP were measured on a Siemens Immulite 2000 XPi system according to the manufactureŕs instructions (Siemens Healthcare Diagnostics, Eschborn, Germany). Plasma beta-2-microglobulin concentrations were measured using a turbidimetric assay (Randox Laboratories, Antrim, United Kingdom). All other laboratory values (i.e., parathormone, hemoglobin, albumin and C-reactive protein) were extracted from the patients' medical records and averaged from the available values of the previous year (4–12 values).

### Clinical data

From each patient data on residual diuresis (measured by 24 h urine collection), single pool Kt/V (mean of last 4 values), interdialytic weight gain, predialytic systolic and diastolic blood pressure (means from the last 12 values beginning from the first blood draw, resp.), dialysis access and membrane, time on dialysis, blood pump flow and finally shunt flow (measured with a Transonic system, Ithaca, NY, USA) were extracted. The systolic left ventricular (LV) function was classified from available echocardiography examinations.

### Statistical analysis

Three plasma samples were available from 87% of the patients and were averaged to calculate the arithmetic mean without excluding possible outliers. Plasma vasoactive peptide concentrations and continuous clinical data were log10 transformed to approximate normal distribution. The association of the plasma vasoactive peptide concentration with clinical or dialysis-related factors was analyzed by univariate parametric correlation. To identify independent determinants of the plasma vasoactive peptide concentration, multivariate linear regression analyses were performed. Selection of the variables entering the model was derived from forward-stepwise multiple linear regression, and all variables with a p-value<0.20 were subsequently included in the multivariate linear regression models. Finally, the residuals of each model were tested for normality. The follow-up period started on the first day of blood draw and lasted until October 26^th^, 2013. Causes of death were classified according to the best knowledge of each particular case. Cardiovascular death was considered as sudden death (most probably circulatory or cardiac arrest) and death due to a cardiovascular event or disease (coronary artery disease, hypertensive crisis, stroke, peripheral artery disease). The averaged values of the deceased patients were compared to those from the surviving patients using a t-Test or Wilcoxońs test. Kaplan-Meier curves were generated after stratification into tertiles of the variable according to its distribution. Survival curves were compared using the log-rank test. Univariate and multivariate proportional hazards were calculated using the Cox regression analysis. Data analysis was performed using the statistical software package JMP 10.0.1 (SAS Institute, Cary, NC).

## Results

### Patients

From a total of n = 250 available patients treated in the participating centers, 239 patients (95.6%) were included in the study. 6 patients declined written informed consent, 2 died within the period of blood sampling, 2 had cardiac amyloidosis and one patient was only recently initiated to dialysis. The characteristics of the study cohort are given in table 1.

**Table 1:** Patient characteristics of the cohort (N = 239).

median age		70 (61; 77) years (n = 239)
gender distribution		64% male (n = 153)/36% female (n = 86)
renal disease		
	diabetic nephropathy	26% (n = 63)
	hypertension	8% (n = 19)
	glomerulonephritis	30% (n = 71)
	polycystic disease	5% (n = 11)
	other/unknown	31% (n = 75)
cardiac comorbidities		
	coronary heart disease/revascularized	31% (n = 74)/19% (n = 31)
	valvular heart disease	26% (n = 61
	atrial fibrillation	23% (n = 55)
	pulmonary hypertension	7% (n = 16)
	AICD carrier	2% (n = 4)
other comorbidities		
	LV dysfunction	25% (n = 59)
	diabetes mellitus	38% (n = 90)
	peripheral vascular disease	33% (n = 80)
	stroke	16% (n = 38)
	vasculitis	3% (n = 8)
	malignoma	14% (n = 34)
	COPD	8% (n = 19)
length of time on dialysis		46 (19; 85) months (n = 239)
duration of dialysis session		4.0 (4.0; 4.5) hours (n = 239)
dialysis access		
	arteriovenous fistula	71% (n = 169)
	PTFE graft	13% (n = 31)
	tunneled catheter	16% (n = 38)
dialysis membrane		
	high-flux	92% (n = 219)
	low-flux	8% (n = 20)
residual diuresis		250 (0; 1000) mL/day (n = 239)
	anuric patients	39% (n = 93)
interdialytic weight gain		1.85 (1.29; 2.47) kg (n = 239)
blood pump speed		300 (280; 340) mL/min (n = 239)
shunt flow		1080 (733; 1475) mL/min (n = 187)
blood pressure		134 (122; 144)/69 (63; 74) mm Hg (n = 239)
singe pool Kt/V		1.55 (1.40; 1.73) (n = 239)
laboratory data		
	hemoglobin	11.5 (11.1; 12.0) g/dL (n = 239)
	C-reactive protein	8.6 (4.6; 15.0) mg/L (n = 239)
	albumin	37.1 (35.4; 39.3) g/L (n = 239)
	parathormone	204 (130; 348) pg/mL (n = 239)
	β2-microglobulin	23.4 (19.4; 25.4) mg/L (n = 239)
	high sensitive troponin T	49 (31; 73) pg/mL (n = 239)
	NT-pro-BNP	4435 (1687; 16228) pg/mL (n = 223)

Values shown are the median and interquartile range. N indicates number of patients from whom data were available.

Abbreviations:

AICD automatic implantable cardioverter-defibrillator, COPD chronic obstructive pulmonary disease, PTFE polytetrafluorethylene, LV left ventricle.

### Plasma concentrations of copeptin, MR-pro-ADM and CT-pro-ET1 in the cohort

**Figure 1:**
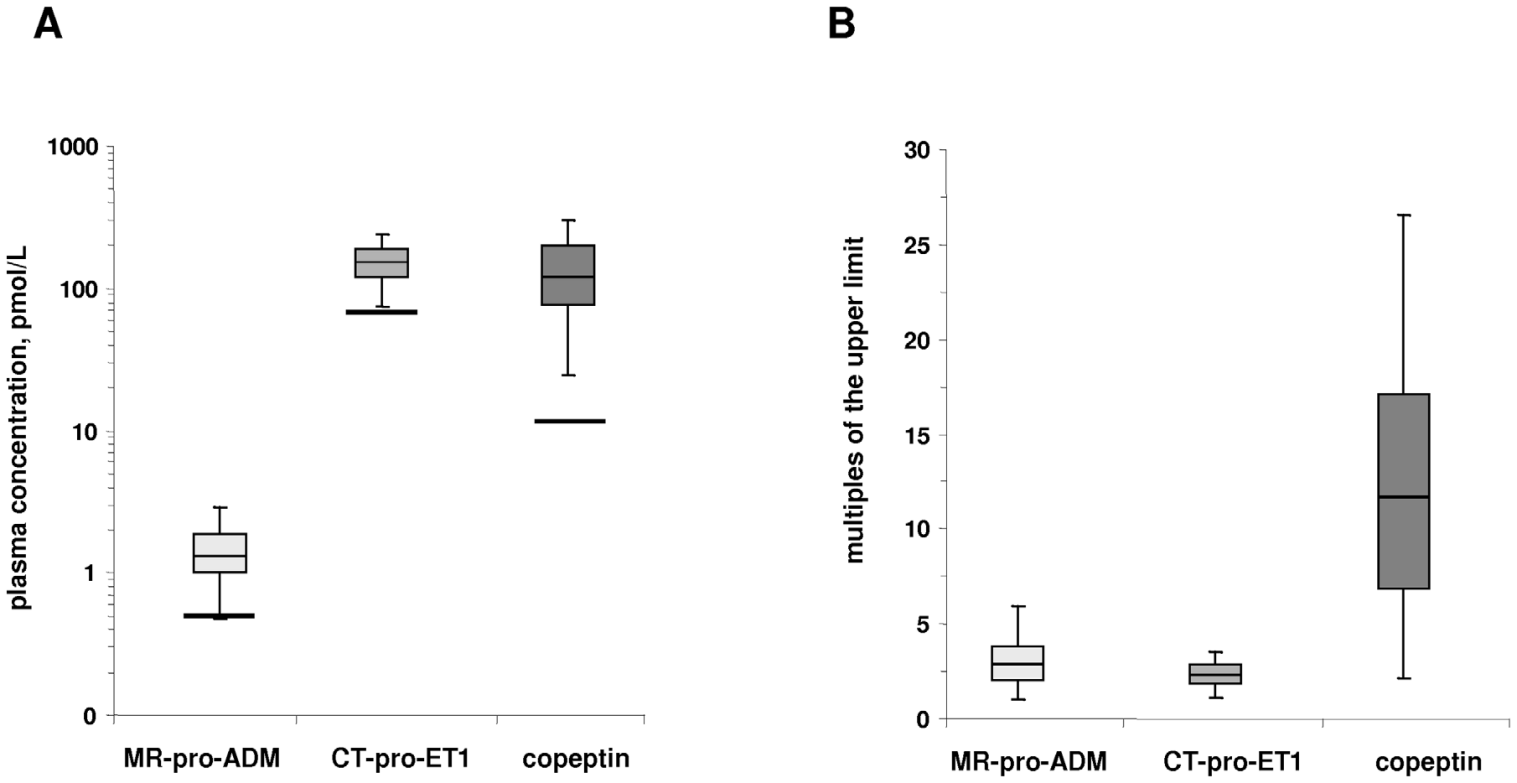
Box-and-Whisker-Plot of the plasma vasoactive peptide concentration indicating median, IQR and the range between the 2.5^th^ and 97.5^th^ percentile (Panel A). Solid lines represent the upper reference limit. Panel B: Box-and-Whisker-Plot of the plasma vasoactive peptide concentration expressed as multiples of the upper reference limit.

In nearly all participating patients, the plasma concentrations of all studied vasoactive peptide fragments were elevated compared to the reference values from healthy persons. The median concentration was 132 pmol/L (IQR 78–192) for copeptin, 1.26 pmol/L (IQR 1.02–1.80) for MR-pro-ADM and 149 pmol/L (IQR 121–181) for CT-pro-ET1 (Figure 1A). Expressed as multiples of the 97.5^th^ percentile taken as the upper end of the reference range obtained from healthy individuals [2]–[4], the copeptin concentration was elevated 11.7-fold (IQR 6.9–16.9) compared to 2.8-fold for MR-pro-ADM (IQR2.1–3.7) and 2.2-fold for CT-pro-ET1 (IQR 1.8–2.7; Figure 1B).

Plasma vasoactive peptide concentrations were moderately correlated to one another, with the highest correlation between MR-pro-ADM and CT-pro-ET1 (0.45, p<0.0001), followed by copeptin and CT-pro-ET1 (0.39, p<0.0001) and MR-pro-ADM and copeptin (0.28, p<0.0001). Within three consecutive samples, the intraindividual variability expressed as the coefficient of variation was the least for CT-pro-ET1 (8±12%), followed by MR-pro-ADM (14±11%) and copeptin (17±14%).

### Univariate analyses

Table 2 lists the results of the univariate correlation analyses of the plasma vasoactive peptide concentration with the collected parameters. MR-pro-ADM, copeptin and CT-pro-ET1 showed a significant positive correlation to time on dialysis and the β2-microglobulin concentration while they were all negatively correlated to residual diuresis. MR-pro-ADM was negatively correlated to systolic blood pressure, while CT-ET1 was positively correlated to diastolic blood pressure. There was weak correlation of all vasoactive peptides to Kt/V and interdialytic weight gain and almost no correlation to cardiac comorbidity except for MR-pro-ADM (table 2).

**Table 2:** Univariate correlations (Pearsońs r) of the plasma vasoactive peptide concentration with general and dialysis-specific parameters (n = 210–239).

	MR-pro-ADM, pmol/L	CT-pro-ET1, pmol/L	copeptin, pmol/L
dialysis-specific factors			
age, y	n.s.	−0.24 ^***^	n.s.
time on dialysis, months	0.34 ^***^	0.33 ^***^	0.30 ^***^
residual diuresis, ml/day	−0.25 ^***^	−0.23 ^***^	−0.33 ^***^
interdialytic weight gain, kg	0.13 ^*^	0.12 ^#^	0.16 ^*^
shunt flow, ml/min	n.s.	0.26 ^***^	0.13 ^#^
systolic blood pressure, mm Hg	−0.18 ^**^	n.s.	n.s.
diastolic blood pressure, mm Hg	n.s.	0.27 ^***^	n.s.
pulse pressure, mmHg	−0.17 ^**^	−0.25 ^***^	n.s.
duration of a HD session	n.s.	0.17 ^*^	n.s.
blood pump speed, ml/min	n.s.	n.s.	n.s.
laboratory data			
Kt/V	0.11 ^#^	0.25 ^***^	0.11 ^#^
hemoglobin, g/dL	n.s.	n.s.	n.s.
plasma albumin, g/L	n.s.	n.s.	n.s.
C-reactive protein, mg/L	0.13 ^#^	−0.11 ^#^	n.s.
parathormone, pg/mL	n.s.	n.s.	n.s.
β2-microglobulin, mg/dL	0.54 ^***^	0.57 ^**^	0.33 ^***^
troponin T, pg/mL	0.26 ^***^	n.s.	0.26 ^***^
NT-pro-BNP, pg/mL	0.42 ^***^	0.24 ^**^	0.24 ^***^
comorbidity (1 = yes)			
systolic LV dysfunction	0.17 ^*^	n.s.	0.13 ^#^
valvular disease	0.28 ^***^	0.17 ^**^	0.18 ^**^
atrial fibrillation	0.35 ^***^	n.s.	n.s.
pulmonary hypertension	0.17 ^**^	n.s.	n.s.
peripheral vascular disease	0.21 ^**^	n.s.	n.s.
diabetes mellitus	n.s.	−0.20 ^***^	n.s.

^#^ p<0.10, ^*^ p<0.05, ^**^ p<0.01, ^***^ p<0.001, n.s.  =  not significant (p>0.10).

### Multivariate analyses

To analyze the independent determinants of the plasma vasoactive peptide concentration, a multivariate linear regression model was performed with a stepwise forward approach based on a p-value of <0.20 and all covariates from table 2. Table 3 lists the resulting multivariate models. Plasma β2-microglobulin concentration was the strongest independent determinant of the plasma vasoactive peptide concentration. The modeling was best for MR-pro-ADM and CT-pro-ET1 with an adjusted r^2^ value of 0.48 and 0.41, respectively, and poor for copeptin (r^2^ = 0.18).

**Table 3:** Independent factors determining the plasma vasoactive peptide concentration by multivariate linear modeling (n = 155–201).

	covariate	estimate ± SD	standardized estimate ± SD	p-value
**MR-pro-ADM R^2^ = 0.48**	y-intercept	−0.85±0.41	0.13±0.01	
	β2-microglobulin, mg/L	0.97±0.10	0.28±0.03	<0.0001
	duration of a HD session	0.64±0.23	0.13±0.05	0.0069
	atrial fibrillation (1 = yes)	0.13±0.02	0.07±0.01	<0.0001
	systolic blood pressure, mmHg	−0.42±0.17	−0.07±0.03	0.0149
	NT-proBNP, pg/ml	0.03±0.02	0.05±0.03	0.0933
	interdialytic weight gain, kg	−0.03±0.02	−0.04±0.02	0.1196
	peripheral vascular disease (1 = yes)	0.06±0.02	0.03±0.01	0.0061
	valvular disease (1 = yes)	0.03±0.02	0.01±0.01	0.2180
**CT-pro-ET1 R^2^ = 0.41**	y-intercept	1.43±0.40	2.17±0.01	
	β2-microglobulin, mg/L	0.56±0.07	0.16±0.02	<0.0001
	NT-proBNP, pg/ml	0.05±0.01	0.07±0.02	0.0009
	age, y	−0.25±0.08	−0.07±0.02	0.0013
	diastolic blood pressure, mmHg	0.43±0.15	0.08±0.02	0.0035
	systolic blood pressure, mmHg	−0.35±0.14	−0.06±0.02	0.0142
	plasma albumin, g/L	0.16±0.12	0.06±0.05	0.1917
	atrial fibrillation (1 = yes)	−0.01±0.02	−0.01±0.01	0.4668
**copeptin R^2^ = 0.19**	y-intercept	0.74±0.27	2.08±0.02	
	β2-microglobulin, mg/L	0.68±0.20	0.18±0.05	0.0009
	time on dialysis, months	0.15±0.05	0.14±0.04	0.0014
	systolic LV dysfunction (1 = yes)	0.09±0.04	0.04±0.02	0.0292
	parathormone, pg/mL	0.07±0.05	0.09±0.06	0.1156

Note that all continuous parameter were entered into the model after log_10_ transformation.

### Prognostic value

After 3.8 years of follow-up, data from all 239 patients were available (median follow-up time 1378 days [IQR 1325; 1429]). Of those, 86 had died (36%), 15 (6%) had been transplanted and the rest remained on HD (table 4). The concentration of MR-pro-ADM was significantly (p<0.0001) higher in the deceased patients compared to the surviving patients (1.62 pmol/L [IQR 1.19;2.27] vs. 1.26 pmol/L [IQR 0.98;1.66]). The concentrations of CT-pro-ET1 and copeptin were not different in the surviving patients compared to the deceased patients (p = 0.52 and p = 0.07, respectively).

**Table 4:** Follow-up data after 3.8 (IQR 3.6;3,9) years and causes of death.

continued on HD	138 (57.7%)
transplanted	15 (6.3%)
deceased	86 (36.0%)
sudden death	20 (23% of all deaths)
cardiovascular death§	14 (16%)
infection and sepsis	12 (14%)
wasting/cachexia	20 (23%)
malignancy	9 (10%)
others/unknown	11 (13%)

§ Composite of deaths due to coronary artery disease, hypertension, peripheral artery disease.

**Figure 2:**
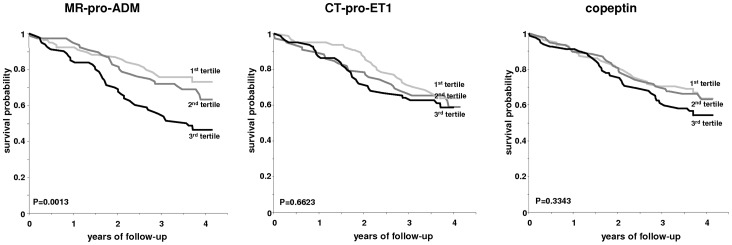
Survival curves of the cohort stratified according to the tertiles (T1, T2 and T3) of MR-pro-ADM (<1.09; 1.09–1.64; >1.64 pmol/L), CT-pro-ET1 (<131; 131–171; >171 pmol/L) and copeptin (<101; 101–171; >171 pmol/L) plasma concentration.

**Figure 3:**
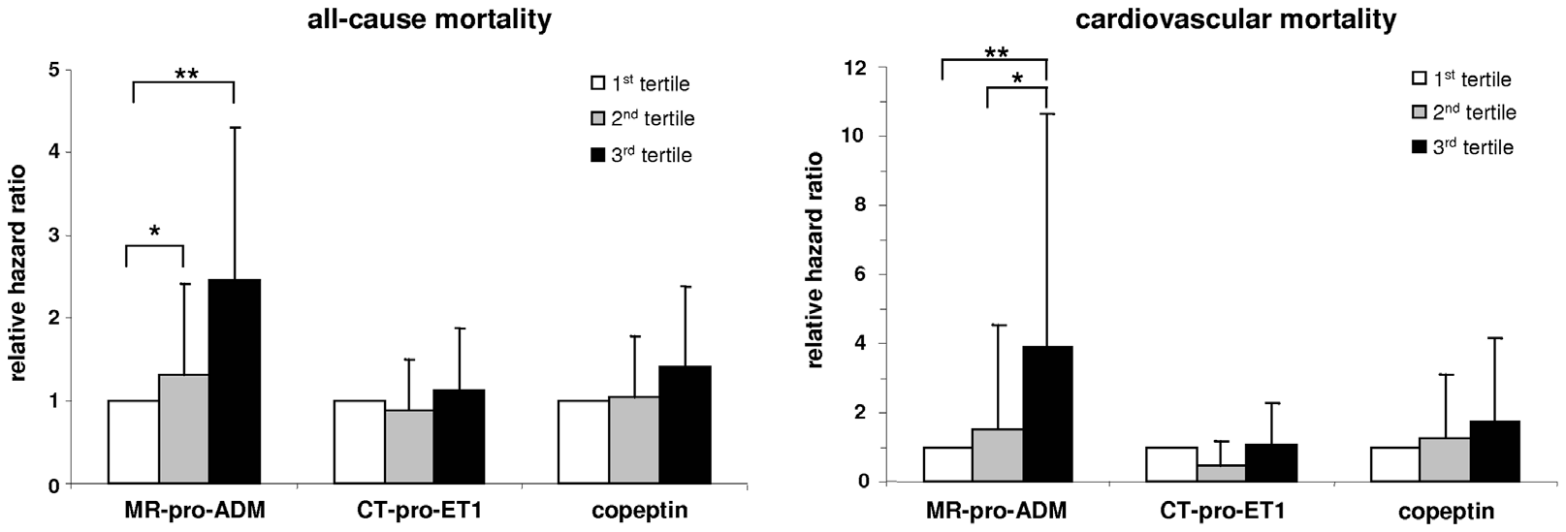
Relative hazard ratios for all-cause mortality and cardiovascular mortality with 95% confidence interval according to the tertiles (T1. T2 and T3) of MR-pro-ADM (<1.09; 1.09–1.64; >1.64 pmol/L), CT-pro-ET1 (<131; 131–171; >171 pmol/L) and copeptin (<101; 101–171; >171 pmol/L) plasma concentration. The risk of the first tertile was set to 1. ^*^ p<0.05, ^**^ p<0.01.

The survival curves stratified for tertiles of vasoactive peptide concentrations are shown in Figure 2, and the relative risk compared to the first tertile is shown in Figure 3. Using the Cox proportional hazard method, plasma MR-pro-ADM concentration was found to be significantly associated with increased all-cause and cardiovascular mortality in a univariate analysis (hazard ratio for a 10-fold increase 9.94 and 34.87, respectively; table 5). These associations were stable in a model adjusted for dialysis-specific factors and finally attenuated in a model adjusted for all prognostic factors. Plasma copeptin concentration was weakly associated with all-cause (p = 0.077) and cardiovascular mortality (p = 0.033) in a univariate model, and lost its associations in multivariate analyses. Plasma CT-pro-ET1 concentration was not associated with a significantly increased hazard ratio, both in univariate and multivariate analyses.

**Table 5:** Hazard ratios (and 95% CIs) for mortality according to the MR-pro-ADM, CT-pro-ET1 and copeptin levels.

	univariate (crude)		multivariate: model 1		multivariate: model 2	
	HR with 95% CI	p-value	HR with 95% CI	p-value	HR with 95% CI	p-value
MR-pro-ADM						
all-cause mortality	9.94 [3.14–31.89]	<0.0001	9.54 [1.81–51.17]	0.0079	3.37 [0.82–14.88]	0.0924
cardio-vascular mortality	34.87 [5.58;217]	0.0001	101.26 [5.47;2317]	0.0017	6.14 [0.66; 71.92]	0.1138
CT-pro-ET1						
all-cause mortality	1.21 [0.25–6.18]	0.8185	3.52 [0.29–43.31]	0.3212	3.10 [0.47–21.97]	0.2427
cardio-vascular mortality	2.27 [0.18;32.63]	0.5293	2.75 [0.28;27.29]	0.3813	5.57 [0.26;138.83]	0.2741
copeptin						
all-cause mortality	2.01 [0.92–4.66]	0.0770	2.37 [0.56–11.58]	0.2492	0.97 [0.39–2.60]	0.9550
cardio-vascular mortality	4.24 [1.11;19.57]	0.0334	5.95 [0.81;63.43]	0.0824	2.62 [0.50;17.61]	0.2669

The hazard ratios are displayed for a one-unit increase in the decadic log-transformed (corresponding to a ten-fold increase) of the plasma MR-pro-ADM, CT-pro-ET1 and copeptin concentration. Cardiovascular mortality was considered as sudden death (most probably circulatory or cardiac arrest) and death due to a cardiovascular event or disease (coronary artery disease, hypertensive crisis, stroke, peripheral artery disease).

Model 1: adjusted for demographics, hemodynamic and dialysis-specific risk factors.

- age.

- gender (male).

- time on dialysis.

- residual diuresis.

- shunt flow.

- vascular access on study enrolment (fistula, graft or catheter).

- systolic and diastolic blood pressure.

- interdialytic weight gain.

- pooled Kt/V.

Model 2: adjusted for all factors associated with mortality (significant difference between deceased and surviving patients).

- age.

- body mass index.

- duration of HD session.

- diastolic blood pressure.

- secondary hyperparathyroidism (represented by parathormone concentration).

- inflammatory status (represented by CRP concentration).

- comorbidities: systolic dysfunction (1 = yes), peripheral vascular disease (1 = yes), coronary artery disease (1 = yes), atrial fibrillation (1 = yes).

## Discussion

This study demonstrates that the plasma MR-pro-ADM concentration is a strong predictor of mortality in hemodialysis patients, whereas copeptin was only weakly and CT-pro-ET1 concentrations not at all associated with mortality. Similar results have been published for MR-pro-ADM in hemodialysis patients by Gouya et al. in 2011 [13], who reported a two-threefold increased hazard ratio for mortality of the upper tertile of the patients compared to the lower tertile. The association of the plasma copeptin concentration with mortality has been previously reported in a study with the cohort of the 4D study (German Diabetes Dialysis Study) [14]. In that large study, copeptin was significantly associated with both all-cause and cardiovascular death, however, we could only demonstrate that copeptin was weakly associated with cardiovascular mortality. The reason for this discrepancy could be related to the studied cohort and statistical power since the 4D study comprised 1241 solely diabetic patients reaching the end point by 37–49%. To our knowledge, the results presented on CT-pro-ET1 in this study are reported for the first time and show that the plasma CT-pro-ET1 concentration is not predictive of mortality in hemodialysis patients. Data on big endothelin 1, the 38 amino acid precursor molecule of endothelin 1, have shown a modest association with mortality in hemodialysis patients, which was lost during multivariate modeling [15]. The CT-pro-ET1 assay used in this study measures a fragment at the C-terminus comprising amino acid residues 168 through 212 of the initial large precursor molecule pre-pro-ET1 (212 amino acids) [4] from which big endothelin 1 (amino acids residues 53–90) and finally endothelin 1 (amino acid residues 53–73) are derived. The reason why the fragment CT-pro-ET1, although elevated in nearly all studied patients, failed to be associated with mortality in contrast to patients with heart failure [16] or patients after acute myocardial infarction [17] remains unclear.

Both endothelin and vasopressin cause vasoconstriction, while adrenomedullin is a peptide with strong vasodilating effects. The association of MR-pro-ADM with mortality suggests that an increased vasodilatory response in hemodialysis patients is prognostically relevant and that low levels of MR-pro-ADM represent a state of less vasoconstriction and better prognosis. The finding of increased cardiovascular mortality with increased copeptin concentrations supports this notion. In addition, it is also conceivable that there is a resistance to the vasodilatory response of MR-pro-ADM in hemodialysis patients leading to increased secretion. In a study with stable kidney transplant recipients, MR-pro-ADM concentrations correlated with the resistance to antihypertensive treatment which suggests increased vasoconstriction in these patients [18]. Studies from Yoshihara et al. found that MR-pro-ADM plasma concentrations reflected volume overload [19]; [20]. In this context, increased MR-pro-ADM plasma concentration can be interpreted as a counterregulatory response similar to the increase in the plasma concentration of the natriuretic peptides which are strong predictors of mortality [11] and also exert vasodilating effects [21]. Physiologically, adrenomedullin mRNA is found in the adrenal gland, heart, kidney and blood vessels and up-regulated by inflammation, hypoxia, oxidative and mechanical shear stress, activation of the renin-angiotensin and the sympathetic nervous system [22]. Pharmacological application of adrenomedullin has shown protective effects in models of renal [23] or heart failure [24]. From this perspective, adrenomedullin can be considered as a marker of stress in cardiovascular disease, and particularly in patients with renal failure who have all the above mentioned features.

Our data show that the plasma concentrations of MR-pro-ADM, CT-pro-ET1 and copeptin were greatly increased in hemodialysis patients, most likely reflecting accumulation. This result was particularly true for copeptin, which was increased about tenfold above the upper limit. This finding indicates that the clearance of these peptide fragments is highly impaired during dialysis-dependent chronic kidney disease, which is typical for low molecular weight proteins. A marker of the latter is β2-microglobulin (molecular weight 11.7 kDa), which was a strong independent predictor of the plasma concentration of all vasoactive peptides studied. On the other hand, modern dialysis practices such as high flux dialyzers or hemodiafiltration can remove significant amounts of low-molecular weight proteins as this was known for β2-microglobulin [25] and also was shown for MR-pro-ADM [19]. In that study the plasma MR-pro-ADM concentration was approximately 25–30% lower after a hemodialysis session. Hence, hemodialysis patients reach a new steady state at a higher level.

The increased plasma concentrations complicate the interpretation of MR-pro-ADM, copeptin and CT-pro-ET1 values, but one has to consider that accumulation can only occur when these peptides are released from their original sites upon biological stimuli, therefore we must assume that there is an increased release of these peptides in hemodialysis patients. The increased plasma concentrations in turn, however, make the interpretation more difficult and only with the aid of studies such as this, clinically meaningful cut-off values can be derived such as the limit of the upper tertile for the prognostic use of MR-pro-ADM (i.e. >1.62 pmol/L). However, more studies are needed to better define cut-off values of the plasma concentration of these peptides for diagnostic and prognostic purposes.

The limitations of the current study might include the incomplete collection of the data relevant to understand the correlation of the studied parameters. In the multivariate regression model, >50% of the variance was not explained by the entered factors, in the case of copeptin even >80%. The study focused on nephrological parameters that are commonly available and accessible during hemodialysis treatment, such as residual diuresis or shunt flow. Furthermore, survival analyses might be limited by the low mortality during the follow-up period.

In conclusion, the fragments of the vasoactive peptides MR-pro-ADM, CT-pro-ET1 and copeptin are largely elevated in hemodialysis patients because of accumulation and, most likely, higher release. Plasma MR-pro-ADM concentration was strongly associated with all-cause and cardiovascular mortality.
